# Five-year results of endovascular aortic repair used according to instructions for use give a good general outcome for abdominal aortic aneurysm

**DOI:** 10.1177/2050312119853434

**Published:** 2019-05-23

**Authors:** Runa G Unsgård, Martin Altreuther, Conrad Lange, Tommy Hammer, Erney Mattsson

**Affiliations:** 1 Department of Radiology, St. Olavs Hospital, Trondheim University Hospital, Trondheim, Norway; 2 Department of Surgery, St. Olavs Hospital, Trondheim University Hospital, Trondheim, Norway; 3 Department of Circulation and Medical Imaging, Norwegian University of Science and Technology, Trondheim, Norway

**Keywords:** Endovascular aortic repair, endovascular, abdominal aortic aneurysm, instructions for use, mortality

## Abstract

**Objectives:**

The primary aim of this study was to investigate the rate of sac enlargement and secondary procedures after 5 years when instructions for use are strictly applied with endovascular aortic repair. The secondary aim was to investigate if strict indications with endovascular aortic repair, rendering more open operations, would change the general outcome of patients with abdominal aortic aneurysm.

**Materials and methods:**

Patients having their abdominal aortic aneurysm procedure in a single institution between 01 January 2002 and 31 December 2006 were included. Indications for endovascular aortic repair were as follows: aortic neck: length 15 mm or more, diameter 32 mm or less and straight configuration; iliac arteries: length > 10 mm, 7.5–20 mm in diameter. Sac enlargement was defined as an increase in diameter of 5 mm or more.

**Results:**

A total of 123 patients were intended to be treated electively with endovascular aortic repair from 2002 to 2007 using Cook Zenith stent grafts. In the same period, 147 patients were treated with elective open repair. At 5 years, 7.3% (N = 9) of the elective intended-to-treat patients with endovascular aortic repair had a sac enlargement. Thirty-five percent of the patients were registered with endoleaks, 13% of the patients had secondary procedures, 12.2% of the patients had early and 6.5% late complications during the follow-up period. Aneurysm rupture was seen in 1.6% of the patients. During the 5-year follow-up period, 34 (27.6%) of the endovascular aortic repair patients died. Five-year mortality for open repair was 23.8%, and 12.2% of the open repair patients had secondary procedures.

**Conclusion:**

Endovascular aortic repair for abdominal aortic aneurysm in accordance with instructions for use gives a low long-term risk for increased diameter and low rate of secondary procedures. There was similar mortality after elective endovascular aortic repair and open repair for abdominal aortic aneurysm. Applying endovascular aortic repair according to instructions for use does not seem to change the general outcome of patients with abdominal aortic aneurysm but improves the outcome with the method.

## Introduction

Over the past few decades, endovascular aortic repair (EVAR) has become the mainstay of treatment for abdominal aortic aneurysm (AAA). The EVAR method of treatment for AAA has been connected with enthusiasm, but there are also indications that EVAR should not be used without regard of specific instructions. Studies have shown a high rate of long-term AAA sac expansion when compliance with device instructions for use (IFU) is low.^1,2^ Rupture risk increases with the size of the aneurysm, which implies that some treated patients will still have an increased risk for rupture.^3,4^ Furthermore, the rate of secondary procedures is still high after EVAR in many studies. At our hospital, the IFU for EVAR have been strictly applied. Our hypothesis was that adhering to IFU for EVAR treatment in Central Norway Health Region in 2002–2007 has led to a low prevalence of expanding aneurysms in the first 5 years after EVAR treatment and did not increase the total mortality of the disease. Therefore, the primary aim of this study was to investigate the rate of sac enlargement and secondary procedures after 5 years when IFU are strictly applied. The secondary aim was to investigate if strict indications with EVAR, rendering more open operations, would change the outcome of the medical service given to patients with AAA.

## Materials and methods

This is an observational study of patients who had their primary elective procedure for AAA between 01 January 2002 and 31 December 2006 in Central Norway Health Region. All patients with AAA in the Central Norway Health Region were evaluated at our institution, which is the only one performing treatment for AAA in the region. The study covers descriptive analyses of information and data registered on EVAR patients and treatment by the Department of Surgery, St. Olav’s Hospital, Trondheim University Hospital.

The indication for AAA repair was an aneurysm diameter over 55 mm in men and over 50 mm in women. Patients over 60 years and with an anatomy according to the IFU for the EVAR device were offered the EVAR treatment. Patients under this age limit who were considered unfit for open surgery were also offered EVAR treatment, as long as the aneurysm anatomy was within IFU. EVAR was used with special emphasis on: proximal aortic neck: length ⩾15 mm, 32 mm ⩽ diameter ⩾ 18 mm and straight configuration (cone-shaped neck only with distal narrowing) and iliac arteries: length > 10 mm and 7.5–20 mm in diameter. The iliac vessels were subject to evaluation of iliac calcification, thrombus and angulation with regard to fixation zones.

Computed tomography (CT) scans were to be taken before EVAR, after 6 months and yearly thereafter. CT scans taken 4.5–6.5 years after EVAR were accepted as 5-year controls in this study. Enlargement of the aneurysm is defined as an increase in diameter of 5 mm or more. Some patients had a follow-up CT at a local hospital, as travel distance is long in the health region. If data of 5-year follow-up were missing, it was formally requested from the local hospital and sent to our institution. Complications were classified and graded according to reporting standards for endovascular aortic aneurysm repair.^5^ Endoleaks were analyzed separately. Secondary procedures include all stent graft-related procedures after initial EVAR in the follow-up period.

The data of the open procedures were obtained from the local NORKAR registry, which is part of the Norwegian registry for vascular surgery.^6^ All patients with AAAs not suitable for EVAR, and fit for open surgery, were treated with the standard open surgical technique.

This is an observational study without comparisons. The main analysis is a descriptive analysis of long-term follow-up of EVAR used according to IFU. In parallel, the outcome of open repair (OR) patients is described without the ambition of a comparison. The reason for the presence of the OR patients is to get an impression of whether the overall mortality rate for the AAA patients would increase with less EVAR being performed following IFU and more open surgery. Since the figures for both the methods are similar, we interpret this as there will be no gain if some of the OR patients would be treated with EVAR outside IFU. A randomized study is required for the comparison of mortality between the EVAR and OR patients. If we were to compare survival between the OR and the EVAR groups, for example with a log rank test, there would be no significant difference in this study. However, without randomization, comparison is not valid.

The data used in this publication are part of a continuous quality follow-up of the health care given. The obligation for hospitals to have such quality control in the health care given is regulated by law in Norway and it is not necessary to have patient consent. This type of data can be used also for publication without approval by the regional ethical committee as long as no extra variables are added and the identity of the individuals is not possible to uncover. The regional ethical committee has read the article and given a formal statement that the data can be published without a specific approval (Supplemental Appendix A).

Results are calculated using an intention-to-treat analysis. The clinical rationale for using intention-to-treat analysis instead of the survival method is the practical setting where the patient requires information about the long-term risks of aneurysm increase and other complications prior to the treatment. The normal approximation confidence interval (CI) for binomial proportion was used on the number of patients with increasing aneurysm size, applying a 95% CI. For testing of statistical significance of the standardized mortality ratio (SMR), a 95% CI was applied.^7^ For calculation of P-value, Fisher’s exact test was used. The mortality rates of the general population were obtained from Statistics Norway.^8^

## Results

A total of 123 patients (107 men and 16 women) were intended to be treated with EVAR and Cook Zenith stent. Three other patients were treated with elective EVAR and Gore Excluder stents; these patients are excluded from the analysis, as they are too few to provide any significant result. The mean age at operation was 74 years (range: 57–85 years). Thirty-one percent of the patients were unfit for open surgery. A total of 147 patients (34 women and 113 men) were treated with elective open repair in the study period. The mean age at operation was 73 years for women (range: 61–86 years) and 70 years for men (range: 46–87 years). Mean aneurysm diameter at operation was 65 mm (range 38–115 mm). The patients excluded from both EVAR and OR treatments were not routinely followed. Comorbidity was as commonly seen in patients with aortic aneurysms (Table 1).

**Table 1.** Patient demographics and risk factors in patients treated with EVAR and OR.

Variable	EVAR, N = 123	OR, N = 147
Male	87%	77%
Age (mean + range)	74 (57–85 years)	71 (46–87 years)
Cardiac disease (ischemic heart disease, valvular or CHF)	60%	44%
Prior stroke/TIA	6.3%	11%
Diabetes	5.8%	0%
Smoking history	41.3%	39.5%
COPD	17%	18.4%
Hypertension	41.7%	49%
Hyperlipidemia	36.2%	NA
Diameter of AAA (mm)		
<50	2	7
50–54	10	22
55–59	48	29
60–69	41	45
70–79	15	30
⩾80	7	14

CHF: congestive heart failure; COPD: chronic obstructive pulmonary disease; AAA: abdominal aortic aneurysm; EVAR: endovascular aortic repair; TIA: transient ischemic attack.

Mean aneurysm diameter for the EVAR patients was 62.5 mm (range: 40–105 mm) (Table 1). Two patients had aneurysm diameter < 50 mm at operation. One had a 40 mm saccular aneurysm, regarded to have high rupture risk. The other patient, with an aneurysm size of 47 mm, was treated with EVAR before having a renal transplant. Three of the 10 elective patients with an aneurysm diameter of 50–54 mm had rapid increase in aneurysm size. The remaining seven patients were treated before the threshold for treatment changed from 50 to 55 mm. From 2003, an AAA diameter of 55 mm for men and 50 mm for women was used as an indication for aneurysm repair, a policy that is in accordance with European Society for Vascular Surgery (ESVS) recommendations.

All elective EVAR patients had aortic aneurysm anatomy according to IFU as described before. In all patients treated with an endovascular procedure, both femoral arteries were explored through a cutdown. In 114 patients an aortobiiliacal stent graft was placed, the remaining eight had an aortouniiliacal stent graft and femorofemoral crossover. The patients were routinely given perioperative heparin, antibiotic prophylaxis with cefalotin and postoperative low-molecular weight heparin until discharge. Independent radiologists measured the sizes of the aneurysms with CT scans before EVAR, 6 months postoperatively and yearly thereafter.

In one patient with severe calcification in the arteries, the introducer could not pass the iliac artery in spite of moderate angulation and calcification, and EVAR was not performed. This patient was still alive at the end of the study period. A total of 122 Cook Zenith stent grafts were implanted.

Mean postoperative length of stay after EVAR was 5 days (standard deviation (SD) 3.2 days). Median follow-up time was 63 months, and total follow-up time was 571.1 person years (Table 2). 7.3% of the patients were lost to follow-up with CT scans, no one was lost to follow-up regarding mortality.

**Table 2.** Perioperative variables of EVAR and OR patients.

Variable	EVAR, N = 123	OR, N = 147
Cook Zenith ABI stent graft	114	
Cook Zenith AUI stent graft and femorofemoral crossover	8	
Dacron prosthesis (Braun Unigraft)		147
Length of stay (SD)	5 days (3.2)	10 (SD 10) Range 0–106 days

ABI: aortobiiliacal; AUI: aortouniiliacal; EVAR: endovascular aortic repair; SD: standard deviation.

### Size measurements

Six patients were followed with ultrasound because of renal impairment, in all these the aneurysm had shrunken at least 5 mm and there was no detectable endoleak on the last follow-up CT.

**Figure 1.**
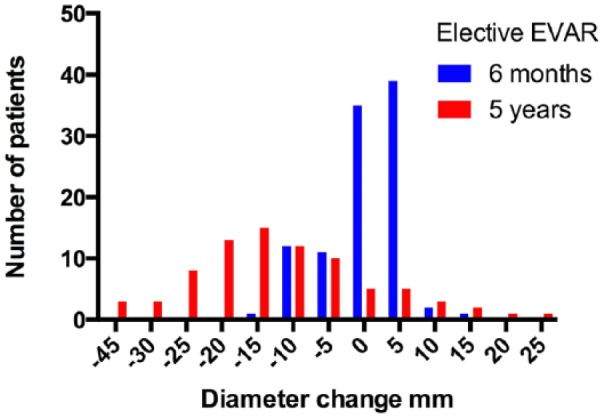
Diameter change in AAA after elective EVAR.

Six patients had increasing aneurysm size at the 6-month CT scan. All these showed shrinking aneurysm size compared to preoperative aneurysm size on the following CT scans. The mean change in diameter after 6 months was −2 mm. After 5 years, the mean change from original aneurysm diameter was −13.7 mm (Figure 1). At 5 years, 7.3% (CI: 2.7%–11.9%) of the intended-to-treat patients (nine of 123) had an increase in aneurysm diameter. Of the 89 patients who had the 5-year CT scan, 10.1% (CI: 3.8%–16.4%) had increase in aneurysm diameter.

### Endoleak

**Figure 2.**
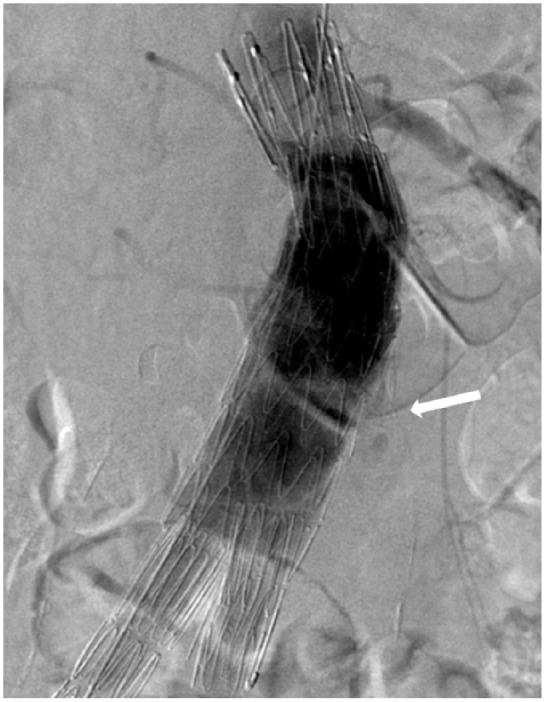
Minimal type I endoleak.

Thirty-five percent of the patients were registered with an endoleak during the follow-up period (Table 3). Five patients had both type I and type II endoleaks, either at the same time or at different times in the follow-up period. 5.7% of the patients had a secondary procedure because of endoleaks. Fourteen patients had an initial minimal type I endoleak (Figure 2), which all resolved spontaneously before the 6-month CT scan. Two major type I endoleaks were treated as soon as possible. Twenty-nine patients had type II endoleaks, of which 13 resolved spontaneously. Seven of these patients had no sac expansion during follow-up and no secondary procedure. Six patients with a type II endoleak had sac expansion, but no secondary procedure because of comorbidity. This resulted in secondary procedures for three patients with type II endoleaks combined with sac expansion. Of the different endoleaks, only endoleak type II leads to increased aneurysmal sacs, which explained seven of the nine sac expansions. The reason for the remaining two sac increases is unknown.

**Table 3.** Registered endoleaks in EVAR patients during 5-year follow-up.

Endoleaks	Total	Resolves spontaneously	Secondary procedure	Size increases at 5 years
Endoleak type I	16	14	2	0
Endoleak type II	29	13	3	7
Endoleak type IIIa	3	–	3	0

EVAR: endovascular aortic repair.

### Complications and secondary procedures

Besides endoleaks, 12.2% of the patients had early complications, mainly deployment related, and 6.5% had late complications (Table 4). Three patients (2.4%) had systemic complications within 30 days. Among the late complications were two aneurysm ruptures (1.6%), both approximately 4 years after EVAR. One of the patients with rupture had a transient early type I endoleak, and later an endoleak type II, but shrinking aneurysm size. When rupture occurred, the patient could not be treated because of severe comorbidity. The second patient survived a type IIIa endoleak and rupture with an endovascular procedure.

**Table 4.** Early and late complications of EVAR and early complications of OR patients.

Complications^a^	Number of EVAR patients with early complications	Number of OR patients with early complications	Number of EVAR patients with late complications
*Deployment related*			
Failed deployment	1 (1)	0	
Access site hematoma	2 (2,3)	0	
Access site infection	2 (1)	1 (0.7%)	
Peripheral macroembolization	1 (3)	0	
Aortic dissection	1 (1)	0	
Access site lymphocele	2 (1)	0	
*Systemic*			
Myocardial infarction	2 (1)		
Myocardial infarction, arrhythmia		19 (12.9%)	
Pulmonary	1 (1)	25 (17%)	
*Device/implant related*			
Buttock claudication	1 (1)		
Postoperative stent graft limb obstruction	2 (2)		5 (2)
Aneurysm rupture	0		2 (2,3)
Stent graft migration	0		1 (2)

EVAR: endovascular aortic repair.

a EVAR patients: classification with grading in parenthesis according to reporting standards for endovascular aortic aneurysm repair.^5^

During follow-up, 16 patients (13%) had altogether 17 secondary procedures (Table 5). Patients with stenosis or kinking had thrombolysis or percutaneous transluminal angioplasty (PTA) and/or femorofemoral crossover.

**Table 5.** Indications for secondary procedures during 5-year follow-up.

Indications for secondary procedures	EVAR	OR
Stenosis	5	3
Kinking	2	
Endoleak type I	2	
Endoleak type II	3	
Endoleak type IIIa	2	
Endoleak type IIIa + rupture	1	
Thrombus in a fibularis	1	
Acute ischemia	1	
Ventral hernia		4
Colon necrosis		1
Abdominal compartment		1
Pseudoaneurysm		1
Postoperative bleeding		3
Wound rupture		2
Infection		2

EVAR: endovascular aortic repair.

One patient with endoleak type I had an endovascular insertion of a palmaz stent, and the other had an endovascular extension of the stent graft. Endoleak type II was treated with coiling and endovascular extension of the stent graft, one patient was treated with injection of onyx. Type IIIa endoleaks were registered in three cases, one of them combined with a ruptured aneurysm as stated under complications. They all had successful procedures with endovascular extension of the stent graft. The patient with a thrombus in a fibularis had an embolectomy on the first postoperative day, and a major adverse event following this ultimately leading to an amputation on the second postoperative day.

Fifteen of 16 patients had successful secondary procedures. The unsuccessful procedure was injection of onyx for a patient with type II endoleak. This patient had shrinking aneurysm size, but was known to later die from a ruptured aneurysm, this patient is described under complications. After secondary procedures nine patients had shrinking aneurysm size, while six patients had an increase in size on the next follow-up CT. One of the patients with secondary procedures was lost to follow-up at 5 years.

12.2% of the OR patients had secondary procedures, where ventral hernia, postoperative bleeding and graft leg stenosis were the most common causes.

### Mortality

During the 30-day postoperative period, there was no mortality. During the 5-year follow-up period, 34 (27.6%) of the patients died. For elective patients, the SMR compared to the general population of the same age was 1.02 for women and 1.25 for men (CI: 0.85–1.78). There was no statistical difference in the mortality rates of the male patients in this study compared to the general population. No conclusions could be drawn for the SMR of women because of the low number of female patients in this study. There were few autopsies performed due to next of kin choice and cultural attitude; therefore, aneurysm-related mortality in this material is difficult to assess. One patient, as mentioned earlier, died from aneurysm rupture. Four of the patients who died during follow-up had an increase in aneurysm diameter on the last follow-up, but none of these patients had clinical indications of aneurysm-related mortality.

### Open repair

Thirty-day mortality was 3.4%, 1-year mortality was 9% and 5-year mortality was 23.8%. There is a tendency to no difference in long-term survival between the EVAR and OR groups, but because of the low number of patients in both study groups, we cannot make any conclusions from this result (P = 0.59).

## Discussion

In this study, adherence to device IFU resulted in a low rate of sac enlargement after 5 years, low rate of complications and secondary procedures. The results of this study also indicate that where IFU have been applied, minimal type I endoleaks can be observed to avoid costly and unnecessary reinterventions. This strategy of this material has not led to unexpected ruptures. Mortality in both EVAR and OR study groups was similar, which indicates that the higher proportion of OR patients does not significantly increase total mortality in patients treated for AAA.

Compliance with IFU gives a low long-term rate of sac expansion after EVAR for AAA, which was 7.3% in this study. In contrast, Schanzer et al.^1^ reported a 41% rate of patients with increased aneurysm size after 5 years when compliance with IFU was low (41.5%–68.9%). Another study with low compliance with IFU found a similar rate of sac expansion after 5 years.^9^ Direct comparison between the studies is difficult due to different study designs, single center versus multicenter and one type of implant versus many different types of implants, but the large difference in sac expansion suggests that compliance with the IFU reduces the long-term rate of aneurysm growth after EVAR. The sac volume was not obtained. Sac volume is an interesting and promising variable to use for the evaluation of AAA changes. The variable is not yet established in clinical practice. This makes it therefore less useful when trying to compare an outcome with that of others where diameter was used. We therefore think that the value and potential impact of our observations are higher when using diameter instead of sac volume.

Initial increase in aneurysm size after 6 months was seen in six cases, which all decreased thereafter. Only one of these patients had an initial small type I endoleak, which resolved spontaneously. All the patients with slightly increasing aneurysm size at 6 months had shrinking aneurysm diameter after 1 year compared to preoperative aneurysm size. This shows that if following IFU, even though patients have unchanged or slightly increased aneurysm diameter at 6 months after EVAR, the treatment might still become successful. One potential explanation is that with better sealing zones minor leakages that are not detected by radiography might cease spontaneously.

The most common side effect of endovascular treatment is different types of endoleaks. A secondary increase in the aneurysmal diameter, as discussed earlier, is the main concern. Thirty-five percent of our patients had an endoleak at some time. This figure is similar to other published results. Schanzer et al. found an overall incidence in 32% of the patients in their study, while Cuypers et al. found an overall incidence of 26% at 18 months in the EUROSTAR registry. The difference is that in our material two-thirds of all type II endoleaks resolved spontaneously or continued without causing aneurysm growth. Minimal initial type I endoleaks in this study were followed and had all resolved spontaneously at 6 months. This rendered only 14.9% of our patients with endoleaks to have a secondary procedure. This can be compared with the EVAR 1 trial in which 33% of the patients had a secondary procedure already within 4 years.^10^ These observations could indicate that initial adherence to IFU gives a better possibility for spontaneous disappearance of endoleaks and a reduced need for secondary procedures on this indication. The reason for this is unclear, but one can speculate that a longer sealing zone is beneficial in the case of an endoleak. Our results with the fates of endoleaks and the diameter over time are in accordance with the findings from a European multicentre registry where patients with temporary endoleaks had a significant decrease in diameter at 6–12 months.^2^

When we include all indications, 13% of our patients had secondary procedures. There are few materials with a comparable follow-up. In the EVAR 1 trial, 20% of the patients had a reintervention after 4 years.^10^ DREAM and OVER trials, with only 2 years of follow-up time, had a reintervention rate of 13% and 13.7%, respectively. The importance to adhere to IFU is opposed by Holt et al.,^9^ who found no difference in reintervention rate for those treated outside IFU (P = 0.136). The estimated reintervention rate in the study was still 24% at 5 years, which is almost the double of our numbers. Again, direct comparison is difficult and the absolute difference in numbers is smaller than for sac expansion.

The number of patients with complications, both systemic and implant related, seems to be similar in this study to what is found in other and larger studies of the EVAR procedure.^11^

Interestingly, a significantly lower survival was seen in the study by Holt et al. when EVAR was applied outside IFU. This latter finding has been supported by other groups.^12^ The overall aim with treatment of AAA is to reduce mortality in those patients having the disease. In our EVAR material, there were no deaths in the 30-day postoperative period, and there was no difference in long-term mortality rate after treatment compared to a matched group of the general population in our country. The potential hidden risk with these good figures with EVAR is a transfer of more AAA patients to be treated with open repair. This could in theory lead to an increased mortality of the AAA patients in general. However, in our data, the long-term survival in the EVAR-treated and OR-treated patients was similar, which supports that applying EVAR according to IFU at an institution does not seem to change the general outcome of patients with AAA. The two patient groups of treatment, OR and EVAR, were not directly comparable and not randomized. At our department, 55% of all elective AAAs were treated with open operation during the 5-year period. Those physically fit and outside IFU for EVAR were offered open repair. There is a tendency of no difference in mortality between the EVAR and OR groups. These findings are in accordance to the literature having no difference in all-cause mortality of patients treated with elective open surgery compared with elective EVAR after 1–2 years.^11,13^ In our case, the patient groups were different, and thereby the methods cannot be compared. The OR group was more fit, and in average 4 years younger. Another recent long-term follow-up study of patients randomized in EVAR and OR groups, however, found a higher aneurysm-related mortality after 8 years for patients treated with EVAR compared to patients treated with open repair.^14^ This study further underlines the importance of following IFU, as more patients could be at a further long-term risk of ruptured AAA if IFU is not followed.

In this study, the OR group consisted mainly of patients outside the IFU of the actual EVAR device, implicating a more challenging anatomy. Our strategy which gives good results for EVAR did not increase the long-term general mortality secondary to OR in our cohort. We therefore do not see any potential advantage in survival if some of the OR patients would have been treated with EVAR outside IFU during this period of time. Furthermore, one can speculate whether the number of patients over time with increased diameter and number of secondary interventions would have become less favorable with the EVAR treatment.

Two of our patients with known aneurysms died within 1 year after open repair for rupture. These individuals had been considered not suitable for endovascular repair and were also unfit for elective open repair. Whether these two patients could have gained anything by applying EVAR outside IFU is difficult to judge.

### Limitations of the study

This study was retrospective with the inborn weaknesses from such a study design. However, the lost to follow-up was very low in comparison to the numbers usually seen with observational studies. Compared to other studies, this study has a small number of patients. This could lead to an inaccurate result; however, we used a calculation of CI with normal approximation to the proportion to correct for random variation. Thus, the number corrected for random variation is under 12% using intention to treat or under 16.5% if using survival calculation. As written in results and discussion, these are good results compared to other studies. A direct comparison of mortality between OR and EVAR is uncertain because of the low number of patients and is therefore not commented upon.

The defined indications for treatment reduce the risk of selection bias. Only one patient treated with EVAR under 60 years of age was considered fit for surgery, but this patient had several risk factors. We therefore evaluate the selection bias as low. This study is retrospective, and the radiologists in this study are independent. We evaluate the measurement bias to be low.

At the time of inquiry and currently, there are no private institutions that perform aortic surgery in Norway. The national health insurance covers the expenditures connected with this type of surgery for all registered citizens; therefore, selection bias with regard to economy is likely low. Two of the elective patients who are registered as lost to follow-up had CTs that show shrinking aneurysm diameter 6.59 and 6.9 years after the initial procedure. If these are included in the result, 5.7% of patients were lost to follow-up. With good results after correcting for chance and an evaluated low level of bias and low number of patients lost to follow-up, we evaluate the study to be valid.

The patients included in the analysis of the EVAR treatment had Cook Zenith stents implanted. This makes the analysis device specific, and the results may not be generalizable to other types of stent grafts. Since this is a single institution report, one should be precautious to extend the findings to other hospitals.

## Conclusion

EVAR for AAA in accordance with IFU for the Zenith device results in low long-term risk for aneurysm growth, low mortality rate and low rate of secondary procedures, compared to other published results. The strict application of EVAR according to IFU at an institution does not seem to change the general outcome of patients with AAA.

## Supplemental Material

Appendix_A_Confirmation_of_approval_1 – Supplemental material for Five-year results of endovascular aortic repair used according to instructions for use give a good general outcome for abdominal aortic aneurysm Click here for additional data file. Supplemental material, Appendix_A_Confirmation_of_approval_1 for Five-year results of endovascular aortic repair used according to instructions for use give a good general outcome for abdominal aortic aneurysm by Runa G Unsgård, Martin Altreuther, Conrad Lange, Tommy Hammer and Erney Mattsson in SAGE Open Medicine
