# Supplementary material for: Five-year results of endovascular aortic repair used according to instructions for use give a good general outcome for abdominal aortic aneurysm
Source: SAGE Open Med. 2019 May 23;7:2050312119853434. doi: 10.1177/2050312119853434 (PMC6535726; doi:10.1177/2050312119853434)
Supplement: Appendix_A_Confirmation_of_approval_1 – Supplemental material for Five-year results of endovascular aortic repair used according to instructions for use give a good general outcome for abdominal aortic aneurysm [file Appendix_A_Confirmation_of_approval_1.pdf]

REK
REGIONALE KOMITEER FOR MEDISINSK OG HELSEFAGLIG FORSKNINGSETIKK

Region:	Executive officer:	Phone number:	Our date:	Our referance:
REC central	Hilde Eikemo	+47 99572363	08.02.2018	2018/262/REK midt

To whom it may concern

This is to confirm that the project "5 year results of EVAR used according to instructions for use gives a good general outcome for AAA" with Erney Mattsson as the principal investigator has been evaluated with the intention to decide if the project needs a full ethical review by the committee. The project was found to be a quality assurance project that does not require a full ethical review according to Norwegian Health Research Act § 2.

Yours sincerely,

Hilde Eikemo
Head of Secretariat
REC central

>
